# Embryonic Temperature Influences the Mucosal Responses of Atlantic Salmon Alevins to a Bacterial Challenge

**DOI:** 10.1007/s10126-024-10386-w

**Published:** 2024-11-19

**Authors:** Muhammad Salman Malik, Alexander Rebl, Erik Burgerhout, Carlo C. Lazado

**Affiliations:** 1 Department of Fish Health, Fisheries and Aquaculture Research, Nofima AS, The Norwegian Institute of Food, 1433 Ås, Norway; 2 Research Institute for Farm Animal Biology (FBN), Wilhelm-Stahl-Allee 2, 18196 Dummerstorf, Germany; 3 Department of Production Biology, Fisheries and Aquaculture Research, Nofima AS, The Norwegian Institute of Food, 9019 Tromsø, Norway

**Keywords:** Yersiniosis, Temperature, Early life stage, Atlantic salmon, Immune response

## Abstract

The present work investigated the effects of embryonic temperature on the responses of Atlantic salmon (*Salmo salar*) alevins to a bacterial challenge using *Yersinia ruckeri* as a model pathogen. Embryos were reared at 4 °C, 6 °C, and 8 °C from fertilization to the eyed-egg stage. Alevins, before the start of feeding, were challenged with the pathogen, and mortality and early immune responses in mucosal organs were assessed. Fish from the 4 °C and 6 °C groups exhibited higher survival probabilities than those from the 8 °C group 72 h post-infection. Mild histopathological changes were observed in the gills and skin across all temperature groups, with bacterial antigen detected in the secondary lamellae of gills and in the skin epithelial and basal layers. Gene expression profiling revealed slightly distinct immune gene expression patterns in low-temperature groups (4 °C and 6 °C) compared to the 8 °C group. *Gelsolin* (*gsn*) expression increased in the skin across all temperature groups at 72 h post-infection. *Claudin* (*cldn4*) and *collagen* (*col1a*) were only upregulated in the skin of the 4 °C group, while *heat shock protein 70* (*hspa1a*) was downregulated in the gills of infected fish at 72 h compared to controls. *Toll-like receptor 13* (*tlr13*) expression increased in infected fish at 24 h compared to controls. In the 6 °C and 8 °C groups, *gsn* expression also increased at 72 h post-infection. *Cldn4* expression increased only in the gills of 8 °C infected fish. This study revealed that low embryonic temperature could influence survival and mucosal immune defences following a bacterial challenge in Atlantic salmon alevins.

**Supplementary Information:**

The online version contains supplementary material available at 10.1007/s10126-024-10386-w.

## Introduction

Temperature is one of the most important environmental factors affecting teleost fish (Burgerhout et al. 2017; Ignatz et al. 2020; Jensen et al. 2015b). As ectotherms, their body temperature regulation is influenced by the temperature of the surrounding water, and key physiological and immunological processes are thermally regulated (Fry 1967; Morvan et al. 1998). Moreover, temperature affects the speed of embryonic development and is associated with phenotypic variations in fish (Ytteborg et al. 2010), including Atlantic salmon (*Salmo salar*) (Gorodilov 1996; Burgerhout et al. 2017; Hayes et al. 1953). Salmon embryos tolerate a range of 0 to 16℃ (Hayes et al. 1953), but increased mortalities are documented at temperatures below 4℃ and above 8℃ (Fraser et al. 2014; Gunnes 1979; Peterson et al. 1977).

To accelerate production, the temperature regimes at commercial salmon hatcheries are often kept at 8℃ (Sommerset et al. 2023). However, higher temperatures can be detrimental to fish health and may pose a risk of heart and vertebral deformities during early life stages in Atlantic salmon (Ytteborg et al. 2010). Changes in embryonic temperature during a relatively short developmental window during embryogenesis (i.e., from fertilization to the eyed-egg stage) have been shown to affect muscle development and growth in salmon (Burgerhout et al. 2017). Additionally, salmon embryos reared at a lower water temperature of 4℃ during this window showed a significantly higher growth potential in seawater stages compared to fish those reared at 8℃ (Macqueen et al. 2008; Burgerhout et al. 2017). These findings highlight that manipulations of embryonic temperature not only affect early development but also shape the growth potential later in life. Besides growth, it is believed that other production parameters, such as health and disease resistance, can also be influenced by temperature during development.

An efficient immune system is responsible for sensing environmental threats and secreting cytokines, chemokines, and inflammatory mediators. However, early influences of temperature variation can modulate host immunity and in some cases, impair the future capacity to cope with environmental stressors such as infectious agents (Zhang et al. 2018). The innate and adaptive immune response depends on the species-specific thermosensitivity and optimum tolerance levels (Morvan et al. 1998). Little is known about the effect of temperature on the developmental plasticity of immune response during embryogenesis. On the other hand, the effects of temperature on immunity, especially in post-larval salmon, have been well documented (Ignatz et al. 2020, 2023; Jensen et al. 2015a, 2015b). Suboptimal temperatures (15 °C instead of 28 °C) during ontogeny suppress *il1b, tnfa, ifng,* and other genes involved in the pro-inflammatory response in zebrafish *(Danio rerio)* (Abram et al. 2017). Low temperature (5 °C) reduces the phagocytic and complement activity in juvenile rainbow trout *(Oncorhynchus mykiss)* (Scharsack and Franke 2022)*.* The skin of turbot (*Scophthalmus maximus*) shows high activity levels of the antimicrobial peptides, lysozyme and hepcidin as well as elevated levels of *immunoglobulin M (IgM),* when exposed to higher temperatures (27 °C) above the thermal tolerance level (16–20 °C) (Huang et al. 2011). Similarly, variation in temperature modulates the expression of genes associated with macrophage activation, mucus secretion, and the pro-inflammatory response in fathead minnows (*Pimephales promelas*) (Wentworth et al. 2018).

The early rearing environment is a strong regulator of immunity and may persistently influence the ability of fish to respond to disease-causing agents later in life. However, there is limited information on how early such robust phenotypes can be observed. This study focuses on rearing temperatures of 4℃, 6℃, and 8℃ from fertilization to the eyed-egg stage of Atlantic salmon. We describe the impact of different embryonic temperature regimes on the disease resistance and immune responses at mucosal sites. In the current study, we employ *Y. ruckeri* as a model pathogen in Atlantic salmon alevins. This pathogen is causative agent of the enteric redmouth disease (ERM) and a relevant model pathogen at this developmental stage.

## Material and Methods

### Ethics Statement

A bacterial challenge experiment was performed on Atlantic salmon alevins before the start of feeding. Therefore, FOTS (*Forsøksdyrforvaltningens tilsyns- og søknadssysteapproval*) approval was not required according to the guidelines of the Norwegian Food Safety Authority (NFSA). According to NFSA’s regulations (*Forsøksdyrforskriften—Section 2.2*), fish species in early developmental stages are exempt from approval requirements for experimental procedures before reaching an independent life stage of self-feeding. Nonetheless, all experimental fish were treated humanely, including euthanasia before sample collection, and humane endpoints were identified and applied. Key personnel in the trial have a FELASA-C certificate.

### Fish and Manipulation of Embryonic Temperature

**Fig. 1:**
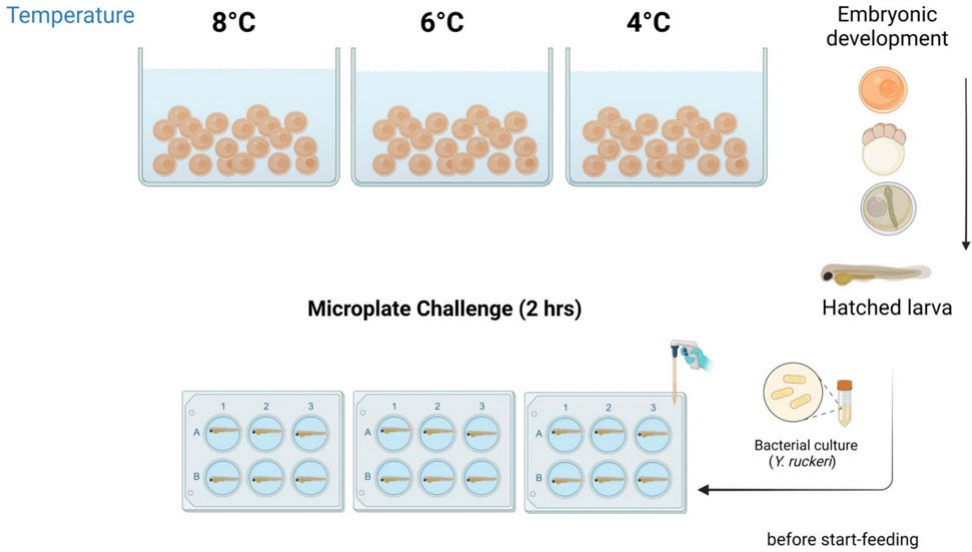
Diagram of experimental setup for *Y. ruckeri* microplate-based challenge trial. Created with BioRender.com

Eggs and milt of 10 females and 10 males (SalmoBreed strain) were purchased from Benchmark Genetics, Norway, and sent to the Aquaculture Research Station in Tromsø, Norway. Ten batches of fertilized eggs (10 male × female crosses) were produced by following the protocol provided by Benchmark. After fertilization, eggs were disinfected using a 1% buffodine solution (Evans Vanodine International PLC, United Kingdom) and divided equally into 9 tanks (130 L each) in a flow-through system, with each tank containing ~ 2000 eggs. Eggs were reared from fertilization until the “eyed-stage” (320-day degrees, DDG) at 3 different water temperatures: 4⁰C, 6℃, and 8⁰C. This developmental period for temperature manipulation was based on previous research showing that during this relatively short time a change in embryonic temperature affected muscle development and growth (Burgerhout et al. 2017; Macqueen et al. 2008). Each group was randomly allocated with 3 replicate tanks. Eggs were monitored daily, and dead eggs were discarded. Upon reaching the “eyed-stage,” the water temperature of the 4 °C and 6 °C groups was gradually increased to 8 °C (2⁰C/day), which was maintained until start feeding (Fig. 1). As temperature influences the rate of development, all procedures were performed based on day degree. The embryos and alevins were kept under photoperiod of 0L:24D.

### Preparation of the Model Pathogen

*Y. ruckeri* isolate serotype O1 (2014–70 646) was provided by the Norwegian Veterinary Institute (NVI, Harstad, Norway). The bacterial stock in glycerol was revived by streaking onto a blood agar plate, followed by incubation for 20–22 h at 12℃. A single colony was also cultured in brain heart infusion (BHI) liquid media under continuous agitation (150 rpm) at 12℃ for 20 h. The optical density (OD) of the culture was measured at 520 nm (UV-1600PC spectrophotometer, VWR International, USA) and adjusted to 0.5 using sterile BHI media. The final density corresponded to 1 × 10^8^ colony forming units (CFU)/mL based on a previous standard curve for OD and bacteria concentration.

### Microplate-Based Infection

Alevins (~ 814 DDG) were randomly distributed in a 6-well microplate with each well containing a single alevin in 8 ml of freshwater (Fig. 1). The alevins were allowed to acclimate in the tank water (refreshed after 24 h) from the research station for 48 h at 8℃ under a photoperiod of 0L:24D before experimental infection was conducted. All alevins were already at 8 °C before first feeding thus infection was performed in all groups at this temperature. Briefly, the water was replaced with freshwater containing 1 × 10^8^ CFU/mL, and alevins were exposed to the pathogen for 2 h. After the exposure period, alevins were washed and transferred to a new microplate with clean freshwater. The control was an uninfected group that was handled similarly but without the addition of the bacterial inoculum. Water was refreshed after every 24 h for infected and uninfected fish until 72 h post-infection. The plates were placed in an incubator with a constant temperature of 8 °C.

Each treatment was performed in triplicate with 6 alevins per replicate. Tissue sampling was performed at 24 and 72 h after the challenge. Alevins were humanely euthanized by overdose (30 mg/L) of isoeugenol (AQUI-S vet., MSD Animal Health, Norway). For gene expression analysis, skin from the dorsal part of the body and gills were suspended in RNAlater (Ambion) and stored at −20℃ until analysis. For histological and immunohistochemical evaluation, whole alevins were fixed in 10% neutral buffered formalin (NBF) for 36 h, transferred to 70% ethanol, and kept at room temperature until processing.

### Histological Evaluation

Alevins (upper body half towards the head end, n = 9) were decalcified in 10% Titriplex (Sigma-Aldrich), for 48 h prior to histological processing. Dehydration and infiltration were performed in an automated tissue processor (TP1020, Leica Biosystems), followed by paraffin embedding to prepare tissue blocks (Leica EG1150H, Leica Biosystems). Sections with 5-µm thickness were prepared in a rotary microtome and stained with haematoxylin and eosin (HE) through an automated stainer (ST5010, Leica Biosystems). Tissue images were generated by a digital slide scanner (Aperio CS2, Leica Biosystems). Samples were then subjected to histopathological evaluation.

### Immunohistochemistry

Whole alevin sections (n = 2, from each group per sampling point) were mounted on a glass slide (Superfrost + ©, Mentzel, Braunshweig, Germany) and dehydrated at 37℃ for 24 h. Sections were baked at 60℃ for 1 h, followed by deparaffinization in an automated tissue stainer (ST5010, Leica Biosystems, Nussloch, Germany) before transferring them to distilled water. Sections were unmasked using a 1:1 ratio of concentrated trypsin and trypsin buffer (Lot GR3327482-1, Abcam UK) for 15 min. Tris buffered saline (TBS) with Triton X-100 (0.1%) was used for permeabilization for 5 min with intermittent shaking. To avoid unspecific staining, sections were blocked by a blocking solution (Bloxall ® Blocking solution; Lot ZJ0817; Vector Laboratories, California, USA) for 10 min at room temperature (RT).

Primary antibody (anti-*Y.ruckeri*, clone 4B12/F8; Lot 0Y7381MS, Ref FM-050AW; Ango, CA, USA) was diluted (1:2000) in TBS with 5% bovine serum albumin (BSA) and incubated for 60 min at RT. After washing with TBS-tween 20 (0.1%) 3 times, secondary antibody (Envision^+^ System- HRP, labelled polymer, anti-mouse; Ref K4001, Lot 11462074; Dako, Denmark) was added on sections for 30 min at RT. Substrate chromogen (EnVision FLEX, HRP Magenta Substrate, Chromogen System; Ref GV925, Lot 41424219; Dako, Denmark) was added for 10 min at RT to generate colour reaction followed by washing with distilled water. Sections were counterstained with Mayer’s Haematoxylin, Lillie’s Modification, Histological Staining Reagent (Ref S3309, Lot 11401568; Dako, Denmark) for 2 min and mounted with VectaMount® AQ Aqueous Mounting Medium (H5501, Vector Laboratories Inc. CA, USA). Scanning was done using a digital slide scanner (Aperio CS2, Leica Biosystems, Illinois, USA).

### Gene Expression Profiling

A panel of 42 carefully selected primer pairs was used to profile the gene expression in the gills and skin of infected versus uninfected alevins (Supplementary File 1). Twenty-nine primers for this panel were adapted from four studies on bacterial infections in Atlantic salmon including *Y. ruckeri*, *Moritella viscosa, Renibacterium salmoninarum* (Bridle et al. 2011; Carvalho et al. 2020; Eslamloo et al. 2020; Rozas-Serri et al. 2020). A further 13 primer pairs originate from a previous report based on more than 100 transcriptional studies on immune-challenged salmonid fish to identify robust markers for immunocompetence (Krasnov et al. 2020). Primers for *ribosomal protein S20* (*rps20)* and *elongation factor-1 (ef1ab)* were used to quantify the expression of two reference genes (Løvoll et al. 2011).

Total RNA from the gills and skin of alevins after bacterial challenge were isolated using an Agencourt RNAdvance™ Tissue Total RNA Purification Kit (Beckman Coulter., CA, USA). RNA purity and quantity were determined by a NanoDrop 8000 Spectrophotometer (ThermoFischer Scientific, USA). The integrity of a selected number of samples was analysed in BioAnalyzer™ RNA 6000 Nano kit (Agilent Technology Inc., Santa Clara, CA, USA). Six 48.48 gene-expression biochips (Standard BioTools) were used to profile the expression of the above primer panel in a total of 252 samples (n = 9). First, the total RNA was adjusted to a concentration of 5 ng/µL and reverse-transcribed in 1 µL (42 °C, 30 min) using Reverse Transcription Master Mix (Standard BioTools). The resulting cDNA aliquots were mixed with primers (100 µM) and PreAmp master mix (Standard BioTools) and individually preamplified in 13 cycles (95 °C, 15 s; 60 °C, 4 min) in a TAdvanced thermocycler (Biometra).

After the pre-amplification step, exonuclease I (New England BioLabs) was added to degrade single-stranded oligonucleotide primers at 37 °C for 30 min. 43 µL of TE buffer (Sigma) was added to each sample. Each 50-µL cDNA sample was diluted in SsoFast EvaGreen Supermix with Low ROX (Bio-Rad) and 20 × DNA Binding Dye Sample Loading Reagent (Standard BioTools) to produce the sample mixes. After transferring the primer and sample mixes together with one no-template (water) control to the assay and sample inlets, the 48.48-gene expression chips were primed in the MX Controller (Standard BioTools). Finally, multiplex qPCR was conducted in the BioMark HD system (Standard BioTools) by following the manufacturer’s thermal protocol (GE Fast 48.48 PCR + Melt v2.pcl). The gene expression data were retrieved as Cq values using Fluidigm Real-Time PCR analysis software v. 4.5.2 (Standard BioTools) and normalized against the reference gene with the least standard deviation, *rps20* (Supplementary Fig. S1).

### Statistical Analysis

All the graphical layouts for statistical analysis were performed with GraphPad Prism version 9.0 (Graphpad Software Inc., La Jolla, CA, USA). Survival data were analysed using Kaplan–Meier survival estimator curves (*p*-value < 0.03). Multiple comparisons were done for significant gene expression analysis using two-way ANOVA in terms of normalized copy numbers of target genes in comparison to the expression level in uninfected fish (control) from all temperature groups at each timepoint. The threshold for statistical significance was set as *p* ≤ 0.05. Heat maps were generated using the “pheatmap” package in R studio (bioconductor; https://www.bioconductor.org/).

## Results

### Survival after Bacterial Infection

**Fig. 2:**
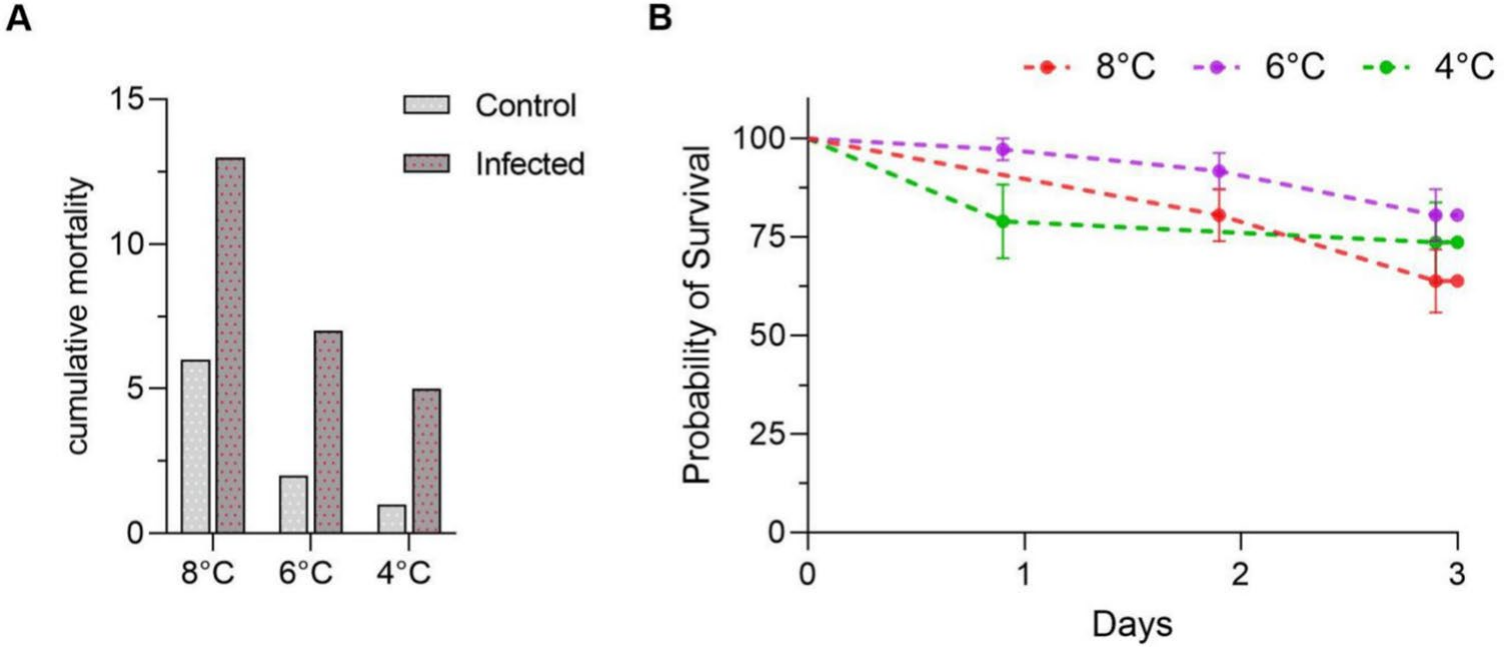
**A** Cumulative mortality in all temperature groups in control and infected plates after 72 h. **B** Kaplan–Meier survival estimator curves showing probability of survival (%) in 8°C (red), 6°C (purple), and 4°C (green) groups with respect to days post challenge. Survival data were analysed using Kaplan–Meier survival estimator curves (p-value < 0.03)

Alevins with different embryonic temperature histories varied in survival 72 h after the challenge with *Y. ruckeri*. The 8 °C group was affected from higher cumulative mortality (n = 13 deaths/36 survivors) compared to the 6 °C (n = 7/36) and 4 °C (n = 5/19) groups (Fig. 2A). Some alevins from the 4 °C group died after transport prior to the challenge, which reduced the number of alevins exposed to *Y. ruckeri* infection in this group (data not shown). The 6 °C group fish had relatively higher survival probability compared to other groups, albeit not statistically significant. There was a difference of 16.69% between the 6 °C (80.56%) and 8 °C (63.89%) groups (*p*-value < 0.11) (Fig. 2B). The control groups exposed to 4 °C and 6 °C had fewer mortalities compared to the 8 °C control group.

### Histological Examination of Gills and Skin from Infected vVrsus Uninfected Alevins

**Fig. 3:**
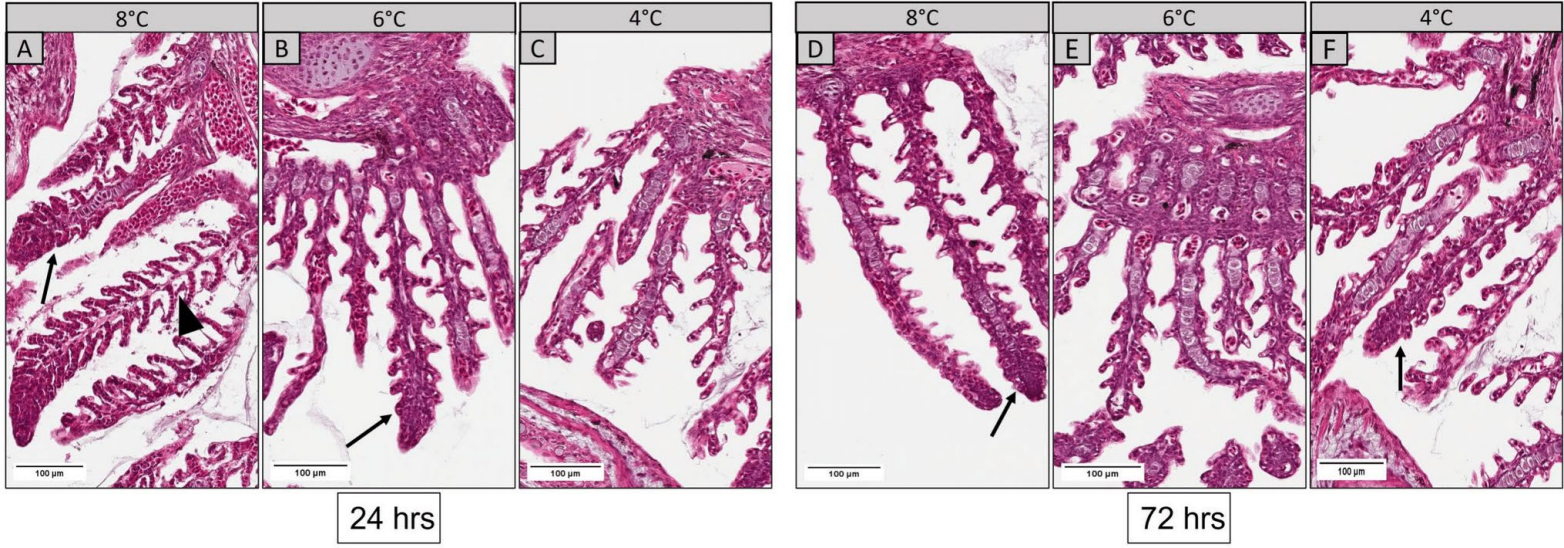
Representative histological sections of the gills from 8°C, 6°C, and 4°C groups at 24 h (A,B,C) and 72 h (D,E,F) after infection with *Y. ruckeri*. Sections were stained with H&E. A few non-specific histopathological changes were observed in different groups, such as lamellar fusion (arrows) and epithelial lifting in secondary lamellae (arrowhead) (scale bar = 100 µm)

**Fig. 4:**
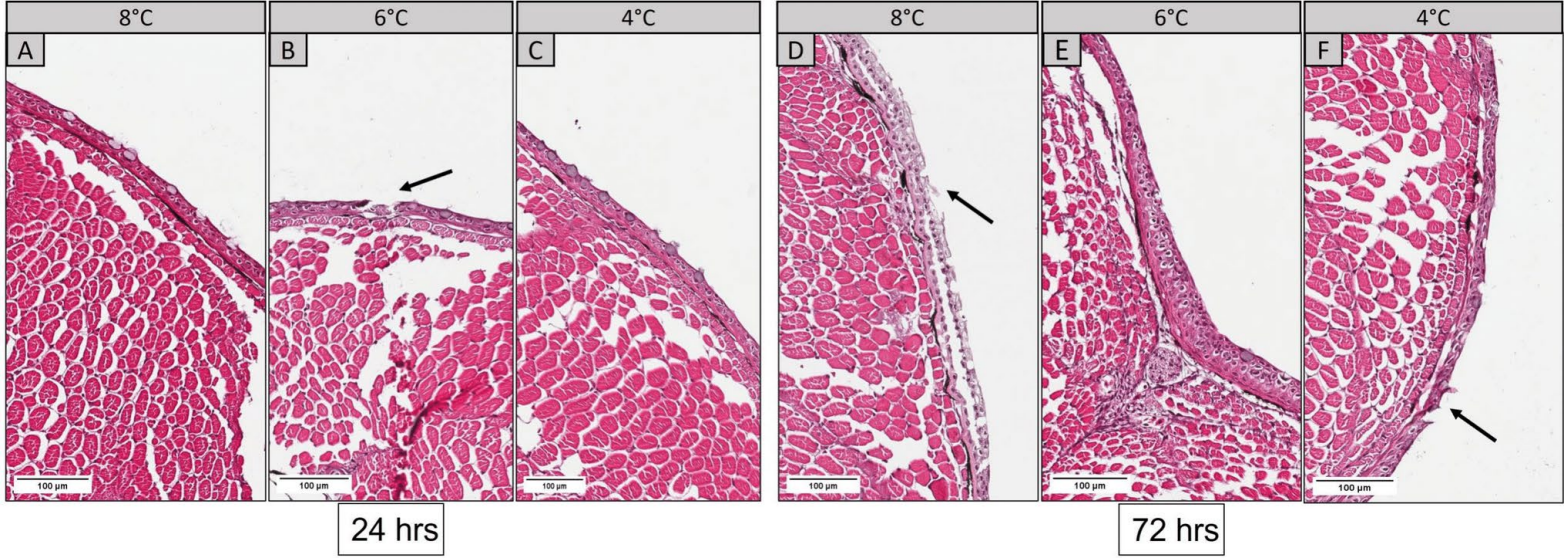
Representative histological sections of the skin from 8 °C, 6 °C, and 4 °C groups at 24 h **A**,**B**,**C** and 72 h **D**,**E**,**F** after infection with *Y. ruckeri*. Few disrupted epithelial surfaces were observed (arrows) at various regions in all temperature groups whereas the *stratum spongiosum* layer was intact (scale bar = 100 µm)

Overall, no major histopathological changes were observed in the gills and skin at 24 h and 72 h after *Y. ruckeri* challenge in all temperature groups. Gills from the 6 °C and 4 °C groups showed normal filaments with intact lamellae but occasional cases of fused lamellae, especially in the 6°C group at 24 h post infection (Fig. 3). Indications of epithelial lifting were observed in the gills of some individuals from the 8 °C group (Fig. 3A). After 72 h, structurally defined primary and secondary lamellae were visible with lamellar fusion rarely occurring in the 8 °C (Fig. 3D) and 4 °C (Fig. 3F) groups.

Similarly, no major histopathological changes were observed in the skin apart from a few epidermal disruptions reflected by a rough outer epidermal layer (Fig. 4). After 24 h, these variations were present in the 6 °C group, but not common in other groups (Fig. 4B). A few areas had loosely detached epidermis after 72 h in the 8 °C group (Fig. 4D) and occasional disruptions and pits in outer skin surface in 4 °C group (Fig. 4F). However, these abnormalities barely occurred in the skin of the 6 °C group after 72 h. Control groups showed smooth and well-defined epithelial structures (Supplementary Fig. S2).

### Immunohistochemical (IHC) Detection and Localization of Y. Ruckeri

**Fig. 5:**
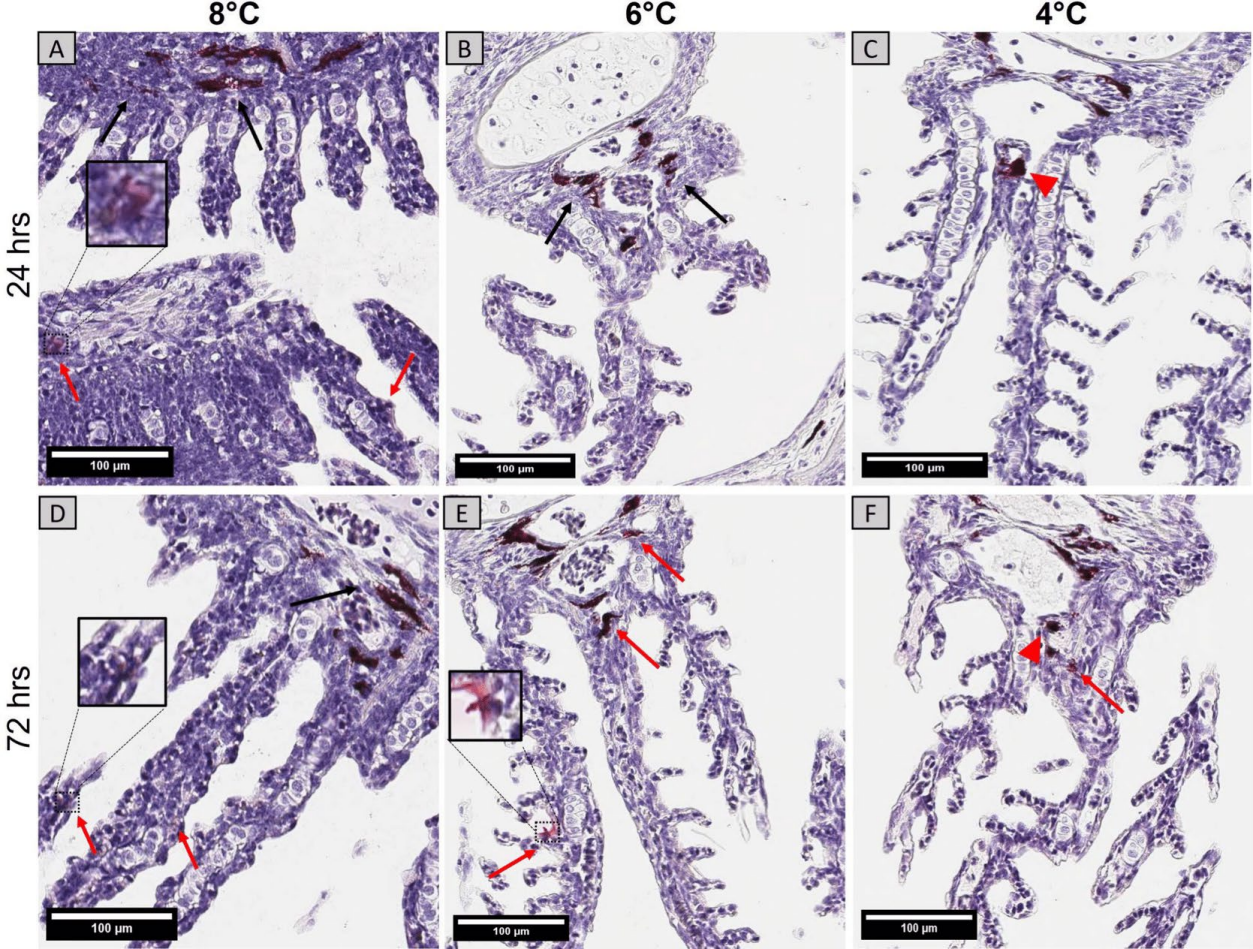
IHC detection of *Y. ruckeri* in the gills of 8°C, 6°C, and 4°C groups at 24 h **A**,**B**,**C** and 72 h **D**,**E**,**F** after challenge. Arrows show positively stained (magenta) cells with bacterial antigen in secondary lamellae (red arrows), around gill arch area (black arrows), and near interbranchial lymphoid tissue (ILT) (red arrowheads) (scale bar = 100 µm)

*Y. ruckeri* was detected in the gills of infected fish from all temperature groups. Mild signals were observed in the secondary lamellae, especially in epithelial surfaces. Bacterial antigen was detected in the periphery of melanized cells in the gill arch and the base of lamellae, and positive staining was noticed near the interbranchial lymphoid tissue (ILT) (Figs. 5C and F). In general, a moderate number of positively stained cells were detected in the gills from all temperature groups 72 h post infection (Fig. 5).

**Fig. 6:**
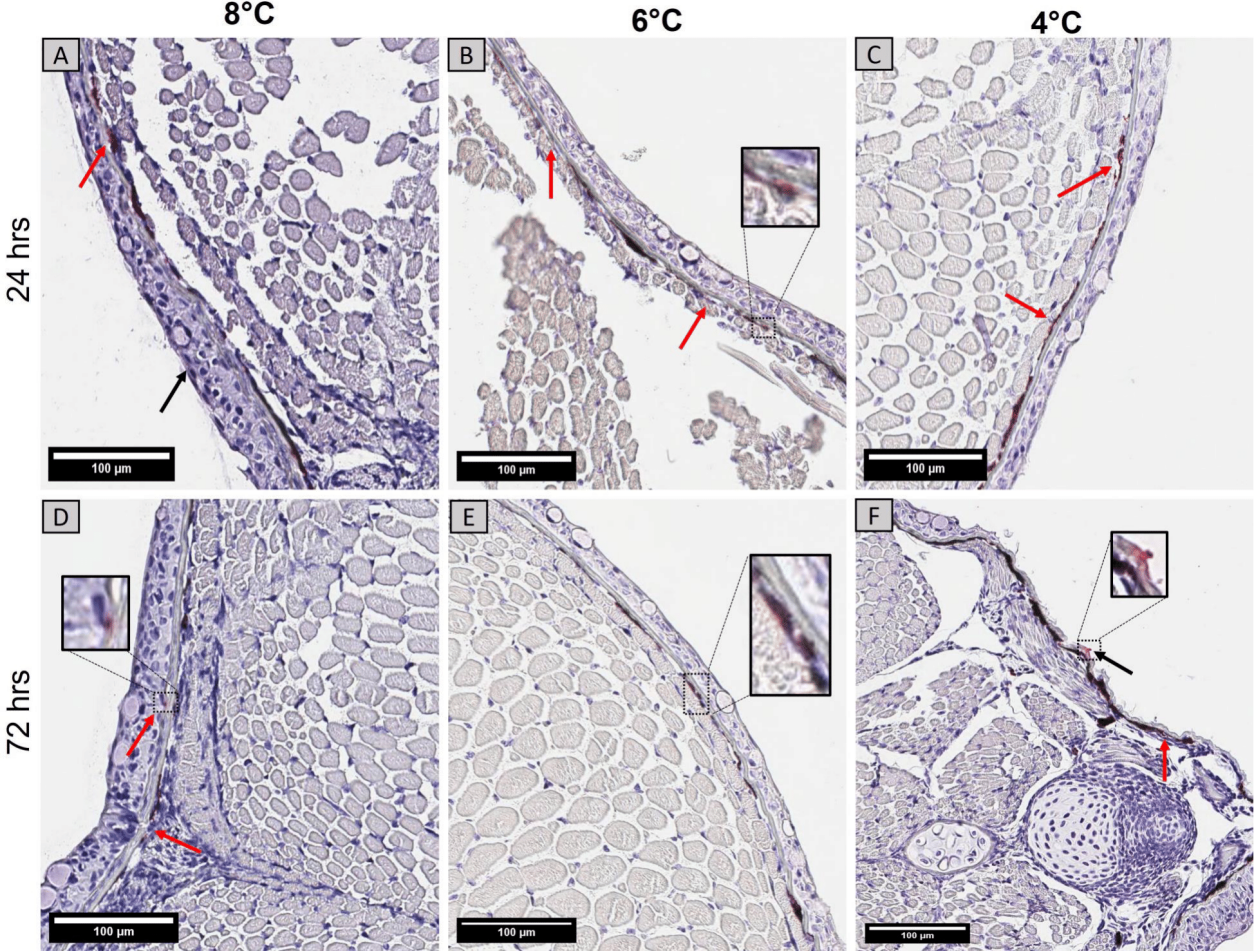
IHC detection of *Y. ruckeri* in the skin of fish from 8°C, 6°C, and 4°C groups at 24 h **A**,**B**,**C** and 72 h **D**,**E**,**F** after challenge. Arrows show positively stained (magenta) cells with bacterial antigen in epidermal (black arrow) and basement layer (red arrow) of skin (scale bar = 100 µm)

In the skin, the bacterial antigen was localized both in the epidermal layer and basement membrane (Fig. 6). Positive staining was noticed in chromatophores in all temperature groups, but no bacterial localization was observed in the skeletal muscle. The immunolabelled bacterial antigen could be detected until 72 h in all temperature groups either in smaller or larger aggregates. Fish from the control groups showed no positively stained cells (Supplementary Figs. S3 and S4).

### Gene Expression Profiles of Infected Versus Uninfected Alevins

**Fig. 7:**
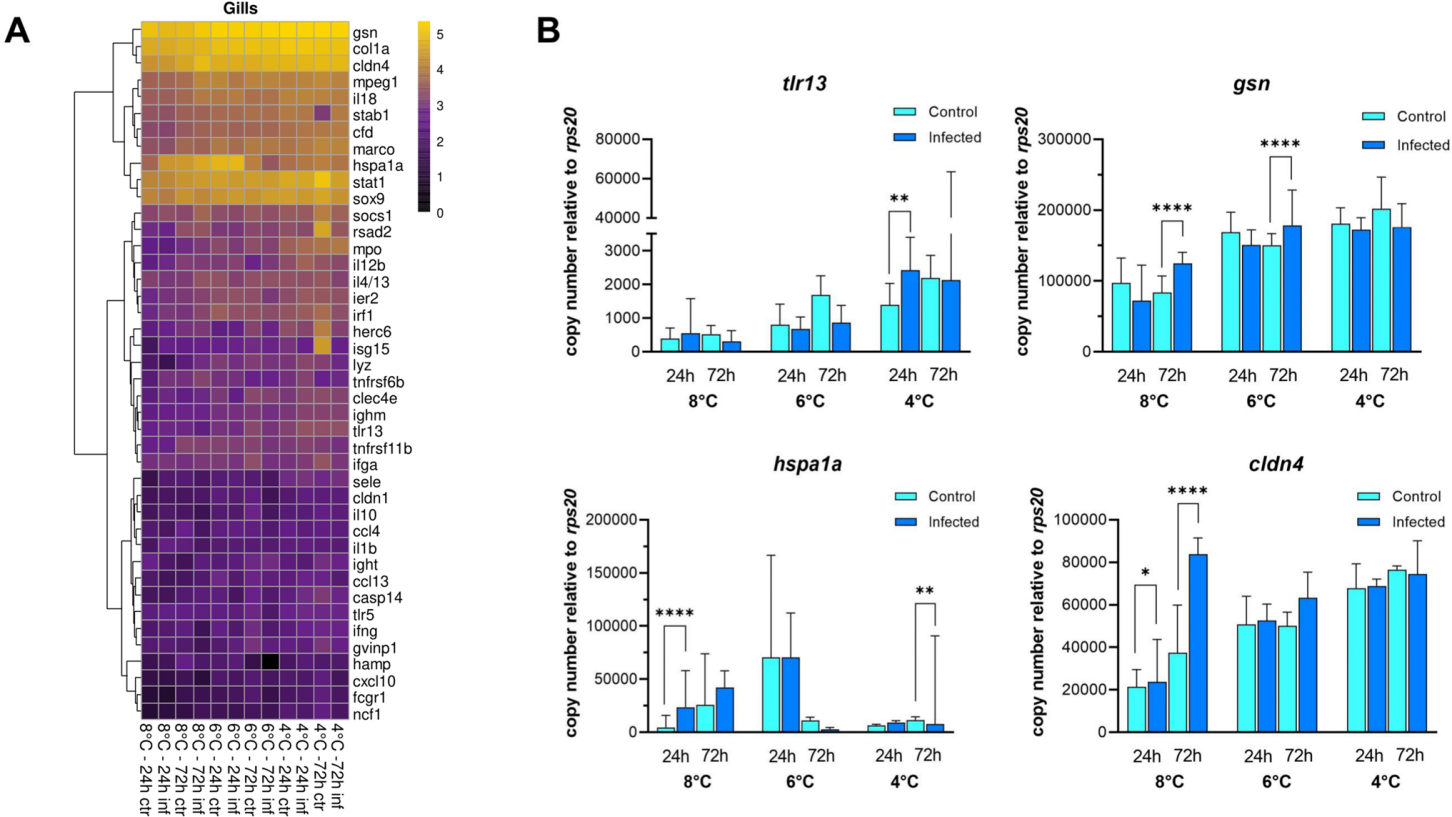
Expression profile of selected genes in the gills. **A** Hierarchical clustering of log_10_-transformed transcript numbers averaged across the individual transcript concentrations in the gills of uninfected (ctr) and infected (inf) salmon alevins 24 h and 72 h after treatment (as indicated below the heatmap). **B** Differentially expressed genes in gills of infected (blue bars) and control alevins (cyan bars). Bars represent the median expression level in each group. Multiple comparisons (Holm-Sidak test) were performed for differential expression analysis. Asterisks indicate significant differences (*p < 0.05, **p < 0.01, **** p < 0.0001)

The gills revealed high expression levels of *gsn*, *col1a*, *cldn4*, *stat1*, *hspa1a*, *sox9,* and *mpeg1* and very low transcript levels of *fcgr1a*, *ncf1*, and *cxcl10* (Fig. 7A). Only four of the 42 selected immune-relevant genes were significantly differentially expressed in the gills between the treatment groups (Fig. 7A). *Toll-like receptor 13 (tlr13)* was upregulated (1.7-fold, *p* < *0.01)* in the infected group compared to the control group in 4 °C group at 24 h after infection (Fig. 7B). At 72 h post infection, the transcript levels of *gelsolin (gsn)* were elevated (1.5 and 1.2-fold, *p* < *0.0001*) in the 8 °C and 6 °C groups, respectively, relative to the control group.

The level of *heat shock 70 (hspa1a)* transcripts significantly increased (5.2-fold, *p* < *0.0001)* at 8 °C compared to the control group at 24 h after infection. However, it was significantly decreased (0.7-fold, *p* < *0.01)* in 4 °C relative to the control group at 72 h after infection. The 6 °C group had negligible *hspa1a* transcript levels after 72 h. Moreover, the *claudin 4 (cldn4)* expression level increased in the infected group relative to the control in the 8 °C group after 24 h (1.1-fold, *p* < *0.05)* and 72 h (2.3-fold, *p* < *0.0001)*.

**Fig. 8:**
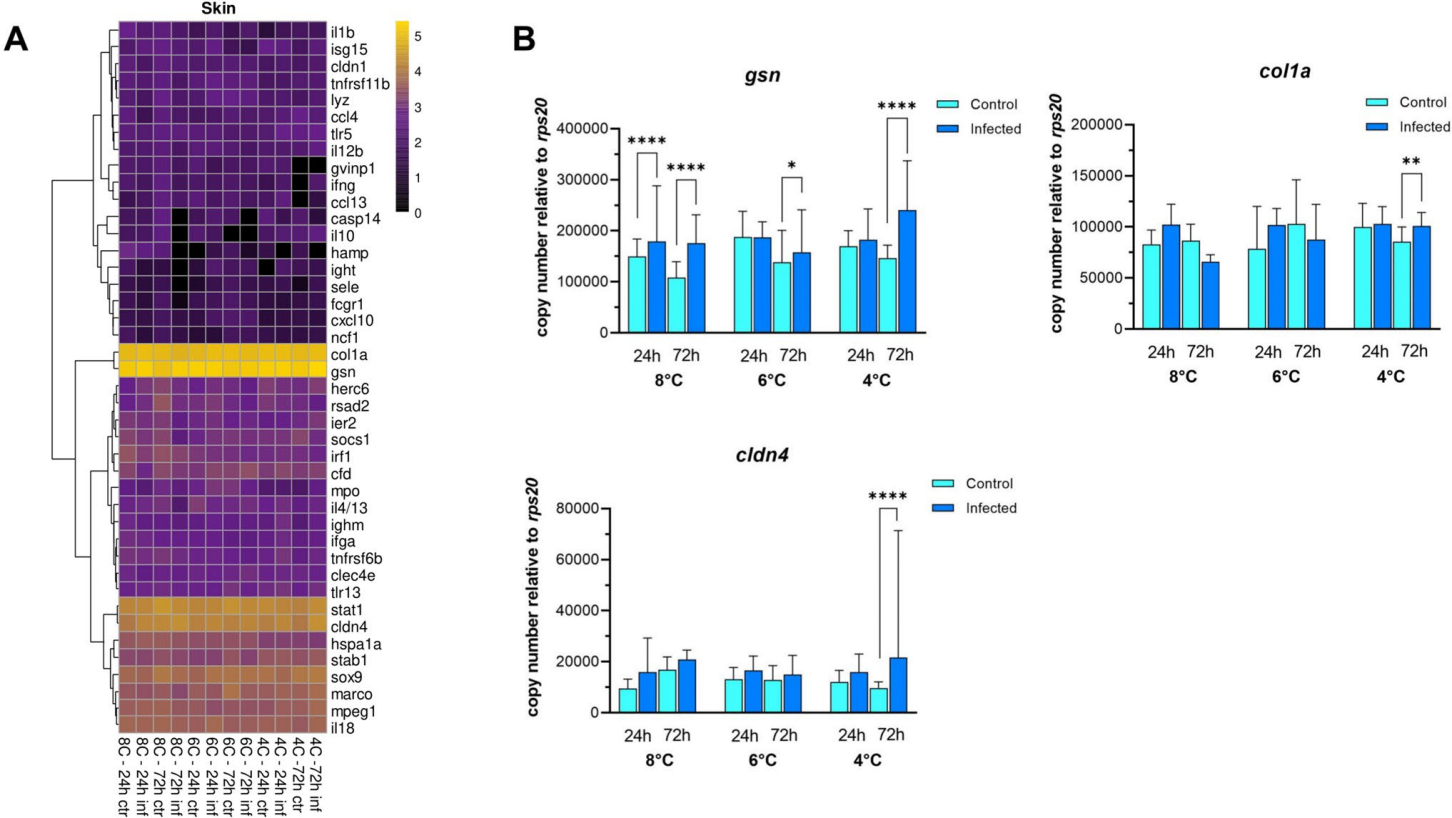
Expression profile of selected genes in the skin. **A** Hierarchical clustering of log_10_-transformed transcript numbers averaged across the individual transcript concentrations in the skin of uninfected (ctr) and infected (inf) salmon alevins at 24 h and 72 h after treatment. **B** Differentially expressed genes in skin of infected (blue bars) and control alevins (cyan bars). Bars represent the median expression level in each group. Multiple comparisons (Holm-Sidak test) were performed for differential expression analysis. Asterisks indicate significant differences (*p < 0.05, **** p < 0.0001)

Strikingly, the tissue-specific expression patterns in the gills and skin shared the same high-level transcripts, including *gsn* and *col1a*, as well as the same low-level transcripts, including *fcgr1a*, *cxcl10*, and *ncf1* (Fig. 8A). Likewise, only a few differences between the groups were detected. At 24 h and 72 h after infection, *gelsolin (gsn)* transcript levels were 1.2- to 1.6-fold higher (with *p* < 0.0001) in all temperature groups compared to the control groups. In addition, *gsn* expression in the infected group was significantly higher than the control at 24 h post-infection in the 8 °C group. The 4 °C group showed the highest increase in the *gsn* level by 1.6-fold (with *p* < 0.0001) at 72 h after infection. At the same timepoint, the levels of *claudin-4* (*cldn4*) and *collagen-I (col1a)* were upregulated by 2.2- and 1.2-fold (with *p* < 0.01) only in the 4 °C group relative to the control groups (Fig. 8B).

## Discussion

Temperature is a strong environmental stimulus that influences nearly every physiological process in poikilothermic vertebrates, including teleost fish (Volkoff and Rønnestad 2020; Ern et al. 2023). In Atlantic salmon, temperature adjustments during early life stages have been shown to be potentially effective in generating phenotypes with enhanced production performance later in life (Burgerhout et al. 2017; Macqueen et al. 2008). In the present study, we investigated the potential impact of embryonic temperature on the mucosal immune response to a bacterial stimulus. We found that lowering the incubation temperature during salmon embryogenesis (from fertilization to the eyed-egg stage) influenced the responses against *Y. ruckeri.* These distinctions were observed as early as before the first feeding. In particular, alevins from the lower temperature groups (4 °C and 6 °C) had a higher survival rate than alevins from the 8 °C group. In addition, low-temperature groups exhibited distinct molecular responses following infection with *Y. ruckeri* relative to the 8 °C group.

### Embryonic Temperature Effects on Survival of Alevins after a Bacterial Challenge

Embryonic development exhibits temperature-dependent plasticity that can either boost or impair fish performance (Scott and Johnston 2012). Albeit not showing strong statistical differences, survival after a bacterial challenge in the lower temperature groups (4 °C and 6 °C) appeared to have of higher survival tendencies than in the group reared at 8 °C. The present study suggests that rearing salmon at temperatures below those often used in the industry might improve disease resistance even before the first feeding. It is likely that temperature histories might have changed the susceptibility of the alevins to *Y. ruckeri,* associated with different survival rates among the temperature groups.

Mucosal barriers on the gills and skin of teleost fish play an important role in preventing pathogen adherence and subsequent entry into the host body (Cabillon & Lazado 2019, Scott and Johnston 2012). Gills have been suggested as the main entry point for *Y. ruckeri*. Upon a successful breach of the mucosal barrier, bacteria proliferate in various organs, such as the spleen, kidneys, and gut, thus causing systemic infection (Tobback et al. 2009). It has also been demonstrated that the lateral canal line, dorsal fin, and gastro-intestinal tract can also serve as active uptake portals (Khimmakthong et al. 2013). Histological evaluation indicated that infection did not result in major alterations in the morphological structures of the gills and skin in all temperature groups. We expected limited histological changes in the target organs following infection because the post-infection period was short. Nonetheless, this observation showed that structural mucosal barrier integrity was good in the experimental fish, despite undergoing transport and handling. Immunohistochemical analysis demonstrated the sparse localization of bacterial antigen in secondary lamellae and near the gill arch in all temperature groups at 24 h and 72 h post-challenge. This is consistent with an earlier study on rainbow trout exposed to *Y. ruckeri,* which detected no intact bacterial particles in the gills after 24 h (Khimmakthong et al. 2013). The bacterial antigen was also detected in the ILT region, which is a vital part of gill-associated lymphoid tissue (GIALT) maintaining immune tolerance and defence in the gills (Aas et al. 2017). In the skin, *Y. ruckeri* was more abundant, particularly in the epidermal layer and basement membrane in all temperature groups. Weak reactions in the chromatophores were consistent, which could be related to the classic external pathology of yersiniosis involving darkening of the skin (Pajdak‐Czaus et al. 2019). Chromatophores are responsible for skin pigmentation, which may change due to bacterial infections (Wildgoose 1998; Lazado et al. 2023).

This study only captured a limited number of timepoints after infection and only directed at the early responses to a bacterial challenge. Therefore, future research must be directed at understanding the temporal and spatial contexts of the infection dynamics of the model pathogen in Atlantic salmon, from evasion of mucosal barrier to infection progression in internal organs, leading to systemic infection.

### Thermal Plasticity of Immune Response

Teleost fish are heavily dependent on innate immunity, especially during the early developmental stages, when adaptive immunity is not well developed (Cornet et al. 2020). The overall expression profiles in the skin and gills following infection showed limited changes relative to the control group. The immunohistochemical detection of *Y. ruckeri* in mucosal organs days after infection was an indication that experimental infection had been successfully carried out. There might be several explanations why only limited changes were observed in the expression of the target genes. First, it could be possible that the timepoints selected were not the best time to capture the antimicrobial response. Second, the strain used was isolated from fish at parr stage and was earlier found to trigger antibacterial response in this life stage (Hovda Aas 2022). It could be possible that the life stage in the current study might have influenced this apparent discrepancy in the host–pathogen response. Third, the fish might have experienced transport stress before infection which might have slightly affected the responses of the fish to the pathogen. Nonetheless, the changes observed in several marker genes offer insights into the impacts of embryonic temperature history on the responses following infection.

Unique structures on pathogens are recognized by pattern recognition receptors (PRRs), such as Toll-like receptors (TLRs), which induce a cascade of immune response (Gürtler and Bowie 2013; Medzhitov 2001). Tlr13, which binds to conserved bacterial 23S ribosomal RNA (Li and Chen 2012; Sahoo 2020), was upregulated after *Y. ruckeri* infection in the gills of the 4°C group, where such change was not observed in the two other temperature groups. Further evaluation is needed to determine whether this expression profile is related to the observed higher survival rate at 4 °C following infection.

Stress proteins including heat shock protein 70 (encoded by *hsp70* or *hspa1a*) are considered important markers for inflammation and other specific and non-specific host responses to environmental stressors or infections (Roberts et al. 2010). *Hspa1a* was significantly upregulated in the gills of 8 °C group at 24 h after infection compared to the negligible expression level in 4 °C group. Moreover, after 72 h, *hspa1a* expression was significantly downregulated in the low-temperature group, which may indicate its suppression following bacterial challenge. In fathead minnow (*Pimephales promelas*), low temperature have been reported to induce *hsp70* expression, but it was restricted to the muscles and brain (Roberts et al. 2010). Upregulation of *hsp70* in the head kidney has been documented in other salmonid species after *Y. ruckeri* infection (Fajardo et al. 2022). Mucosal organs (i.e., gills and intestine) in other teleost fish show high expression of *hsp70* when exposed to bacterial infection (Baharloei et al. 2021). Given the detection of the antigen in mucosal organs, one might expect a striking inflammatory response as a classic reaction to the pathogen. However, we observed limited changes in other key inflammatory response markers (e.g., *il1b*) therefore it is difficult to speculate the influence of temperature changes on inflammatory responses following infection. Nonetheless, the changes showed in *hsp70* expression in the two temperature groups after infection indicate that embryonic temperature changes might influence inflammatory responses, but the extent could not be substantially established by the current data.

Extracellular matrix (ECM) proteins regulate antimicrobial and inflammatory responses. The fibrillar collagen (encoded by *col1a*) constitutes a major portion of the ECM that mediates cell receptor functions, enhances tensile strength, regulates cell adhesion, and promotes tissue development (Frantz et al. 2010). Moreover, collagen is important in activating phagocytes in response to lipopolysaccharide (LPS), one of the most well-studied pathogen-associated molecular patterns (Castillo-Briceño et al. 2009). *Col1a* exhibited slightly higher expression in the skin from the 4 °C temperature group at 72 h after infection, whereas a downward trend was noticed for the 8 °C and 6 °C groups. Despite minimal gross pathological changes, a considerable difference in the *col1a* transcription indicates a noticeable potential for tissue regeneration in the low-temperature group.

Structural proteins such as the actin-binding *gelsolin (gsn)* are also present in the ECM and involved in cytoskeleton remodelling and re-epithelialization in damaged tissue (Méré et al. 2005; Wittmann et al. 2018). They are associated with other mucosal proteins that are highly expressed under inflammatory or stress conditions due to disturbed homeostasis and are considered as a marker of health status (Sanahuja and Ibarz 2015). *Y. ruckeri* can target the actin cytoskeleton in the process of cell invasion (Trosky et al. 2008). *Gelsolin* expression was high especially in the skin at 72 h after *Y. ruckeri* infection and was not affected by different temperature history. This indicates that *gelsolin* could be a crucial defence molecule in salmon against *Y. ruckeri*, which is not influenced by temperature history.

Claudins such as claudin-4 (encoded by *cldn4*) are important components of tight junctions, constituting the major molecular structure in epithelial barrier functions. Previous studies demonstrated their crucial involvement in bacterial infection and “fencing” formation between epithelial cells in teleost fish (Deng et al. 2022; Kolosov and Kelly 2020). These junctions act as an ideal target for the translocation of pathogens to invade the host (Paradis et al. 2021). Reduced inflammation of mucosal surfaces in Turbot (*Scophthalmus maximus*) correlates with increased *cldn4* expression resulting in better barrier function (Dai et al. 2020). In the present study, *cldn4* was significantly upregulated in the gills of infected individuals of the 8 °C group at 24 h and 72 h after infection, while *cldn4* was only elevated in the skin of the 4 °C group at 72 h post infection. However, it is worth noting that the basal gene expression of *cldn4* increased in the gills of uninfected alevins from the low-temperature groups (i.e., 4 °C), which masked the difference between the control and infected groups.

## Conclusions

This study has presented evidence of how slight variation in the rearing temperature during the crucial period of embryonic development could result in different phenotypes, which could be observed as early as before the first feeding. The survival data and gene expression analysis of mucosal organs (skin and gills) suggested that the improved survival probability following *Y. ruckeri* infection in low-temperature groups was likely influenced by the regulation of key molecules in barrier functionality and immune defence. Nonetheless, the slight and limited changes observed in the panel of marker genes indicated potential confounding factors influencing the dynamics of mucosal responses to the pathogen. Additional insights are needed to better understand the contribution of production history on the spatial and temporal *Y. ruckeri* infection dynamics in Atlantic salmon.

## Supplementary Information

Below is the link to the electronic supplementary material.

Supplementary file1 (JPG 602 KB)

Supplementary file2 (PNG 10809 KB)

Supplementary file3 (PNG 7487 KB)

Supplementary file4 (PNG 6344 KB)

Supplementary file5 (XLSX 16 KB)

## Data Availability

Data is provided within the supplementary information files. Additional data can be requested from the corresponding author.

## References

[CR1] Aas IB, Austbø L, Falk K, Hordvik I, Koppang EO (2017) The interbranchial lymphoid tissue likely contributes to immune tolerance and defense in the gills of Atlantic salmon. Dev Comp Immunol 76:247–254 28655579 10.1016/j.dci.2017.06.013

[CR2] Abram QH, Dixon B, Katzenback BA (2017) Impacts of low temperature on the teleost immune system. Biology 6:39 29165340 10.3390/biology6040039 PMC5745444

[CR3] Baharloei M, Heidari B, Zamani H, Ghafouri H, Hadavi M (2021) Effects of heat shock protein inducer on Hsp70 gene expression and immune parameters during Streptococcus iniae infection in a Persian sturgeon fry. Faculty of Veterinary Medicine, Urmia University, Urmia, Iran, Veterinary Research Forum 10.30466/vrf.2019.115181.2740 PMC9010848 35529822

[CR4] Bridle A, Nosworthy E, Polinski M, Nowak B (2011) Evidence of an antimicrobial-immunomodulatory role of Atlantic salmon cathelicidins during infection with Yersinia ruckeri. PLoS ONE 6:e23417 21858109 10.1371/journal.pone.0023417 PMC3153500

[CR5] Burgerhout E, Mommens M, Johnsen H, Aunsmo A, Santi N, Andersen Ø (2017) Genetic background and embryonic temperature affect DNA methylation and expression of myogenin and muscle development in Atlantic salmon (Salmo salar). PLoS ONE 12:e0179918 28662198 10.1371/journal.pone.0179918 PMC5491062

[CR6] Cabillon NAR, Lazado CC (2019) Mucosal barrier functions of fish under changing environmental conditions. Fishes 4:2

[CR7] Carvalho LA, Whyte SK, Braden LM, Purcell SL, Manning AJ, Muckle A, Fast MD (2020) Impact of co-infection with Lepeophtheirus salmonis and Moritella viscosa on inflammatory and immune responses of Atlantic salmon (Salmo salar). J Fish Dis 43:459–473 32100325 10.1111/jfd.13144

[CR8] Castillo-Briceño P, Sepulcre MP, Chaves-Pozo E, Meseguer J, García-Ayala A, Mulero V (2009) Collagen regulates the activation of professional phagocytes of the teleost fish gilthead seabream. Mol Immunol 46:1409–1415 19185348 10.1016/j.molimm.2008.12.005

[CR9] Cornet V, Douxfils J, Mandiki SN, Kestemont P (2020) Early-life infection with a bacterial pathogen increases expression levels of innate immunity related genes during adulthood in zebrafish. Dev Comp Immunol 108:103672 32151677 10.1016/j.dci.2020.103672

[CR10] Dai J, Ou W, Yu G, Ai Q, Zhang W, Mai K, Zhang Y (2020) The antimicrobial peptide cecropin AD supplement alleviated soybean meal-induced intestinal inflammation, barrier damage, and microbial dysbiosis in juvenile turbot Scophthalmus Maximus. Front Marine Sci 7:584482

[CR11] Deng F, Wang D, Chen F, Lu T, Li S (2022) Molecular characterization and expression analysis of claudin-4-like in rainbow trout involved in Flavobacterium psychrophilum infection. Fish Shellfish Immunol 130:244–251 36122640 10.1016/j.fsi.2022.09.016

[CR12] Ern R, Andreassen AH, Jutfelt F (2023) Physiological mechanisms of acute upper thermal tolerance in fish. Physiology 38:141–158 36787401 10.1152/physiol.00027.2022

[CR13] Eslamloo K, Caballero-Solares A, Inkpen SM, Emam M, Kumar S, Bouniot C, Avendaño-Herrera R, Jakob E, Rise ML (2020) Transcriptomic profiling of the adaptive and innate immune responses of atlantic salmon to Renibacterium salmoninarum infection. Front Immunol 11:567838 33193341 10.3389/fimmu.2020.567838 PMC7656060

[CR14] Fajardo C, Santos P, Passos R, Vaz M, Azeredo R, Machado M, Fernández-Boo S, Baptista T, Costas B (2022) Functional and molecular immune response of rainbow trout (Oncorhynchus mykiss) following challenge with Yersinia ruckeri. Int J Mol Sci 23:3096 35328519 10.3390/ijms23063096 PMC8948951

[CR15] Frantz C, Stewart KM, Weaver VM (2010) The extracellular matrix at a glance. J Cell Sci 123:4195–4200 21123617 10.1242/jcs.023820 PMC2995612

[CR16] Fraser T, Fleming M, Poppe T, Hansen T, Fjelldal P (2014) The effect of ploidy and incubation temperature on survival and the prevalence of aplasia of the septum transversum in A tlantic salmon, S almo salar L. J Fish Dis 37:189–200 23488808 10.1111/jfd.12091

[CR17] Fry FJ (1967) Responses of vertebrate poikilotherms to temperature. Thermobiology: Academic Press New York, pp 375–409

[CR18] Gorodilov YN (1996) Description of the early ontogeny of the Atlantic salmon, Salmo salar, with a novel system of interval (state) identification. Environ Biol Fishes 47:109–127

[CR19] Gunnes K (1979) Survival and development of Atlantic salmon eggs and fry at three different temperatures. Aquaculture 16:211–218

[CR20] Gürtler C, Bowie AG (2013) Innate immune detection of microbial nucleic acids. Trends Microbiol 21:413–420 23726320 10.1016/j.tim.2013.04.004 PMC3735846

[CR21] Hayes F, Pelluet D, Gorham E (1953) Some effects of temperature on the embryonic development of the salmon (Salmo salar). Can J Zool 31:42–51

[CR22] Hovda Aas L (2022) Mucosal immune responses of atlantic salmon parr following a pathogen breach in a recirculating aquaculture system. Master's thesis, UiT The Arctic University of Norway. https://www.hdl.handle.net/10037/26570

[CR23] Huang ZH, Ma AJ, Wang XA (2011) The immune response of turbot, Scophthalmus maximus (L.), skin to high water temperature. J Fish Dis 34:619–627 21762173 10.1111/j.1365-2761.2011.01275.x

[CR24] Ignatz EH, Braden LM, Benfey TJ, Caballero-Solares A, Hori TS, Runighan CD, Fast MD, Westcott JD, Rise ML (2020) Impact of rearing temperature on the innate antiviral immune response of growth hormone transgenic female triploid Atlantic salmon (*Salmo salar*). Fish Shellfish Immunol 97:656–668 31891812 10.1016/j.fsi.2019.12.081

[CR25] Ignatz EH, Rise ML, Gamperl AK (2023) Impact of stress phenotype, elevated temperature, and bacterin exposure on male Atlantic salmon (S*almo salar*) growth, stress, and immune biomarker gene expression. Physiol Genomics 55:587–605 37746713 10.1152/physiolgenomics.00055.2023

[CR26] Jensen LB, Boltana S, Obach A, Mcgurk C, Waagbø R, Mackenzie S (2015) Investigating the underlying mechanisms of temperature-related skin diseases in Atlantic salmon, *Salmo salar* L., as measured by quantitative histology, skin transcriptomics and composition. J Fish Dis 38:977–992 25272336 10.1111/jfd.12314

[CR27] Jensen LB, Wahli T, Mcgurk C, Eriksen TB, Obach A, Waagbø R, Handler A, Tafalla C (2015) Effect of temperature and diet on wound healing in Atlantic salmon (*Salmo salar* L.). Fish Physiol Biochem 41:1527–1543 26272065 10.1007/s10695-015-0105-2

[CR28] Khimmakthong U, Deshmukh S, Chettri JK, Bojesen AM, Kania PW, Dalsgaard I, Buchmann K (2013) Tissue specific uptake of inactivated and live Yersinia ruckeri in rainbow trout (Oncorhynchus mykiss): visualization by immunohistochemistry and in situ hybridization. Microb Pathog 59:33–41 23583292 10.1016/j.micpath.2013.03.001

[CR29] Kolosov D, Kelly SP (2020) C-type natriuretic peptide regulates the molecular components of the rainbow trout gill epithelium tight junction complex. Peptides 124:170211 31770576 10.1016/j.peptides.2019.170211

[CR30] Krasnov A, Afanasyev S, Nylund S, Rebl A (2020) Multigene expression assay for assessment of the immune status of Atlantic salmon. Genes (Basel) 11:1236. 10.3390/genes11111236 33105610 10.3390/genes11111236 PMC7690445

[CR31] Lazado CC, Iversen M, Johansen L-H, Brenne H, Sundaram AY, Ytteborg E (2023) Nasal responses to elevated temperature and Francisella noatunensis infection in Atlantic cod (Gadus morhua). Genomics 115:110735 37898334 10.1016/j.ygeno.2023.110735

[CR32] Li XD, Chen ZJ (2012) Sequence specific detection of bacterial 23S ribosomal RNA by TLR13. elife 1:e00102 23110254 10.7554/eLife.00102 PMC3482692

[CR33] Løvoll M, Austbø L, Jørgensen JB, Rimstad E, Frost P (2011) Transcription of reference genes used for quantitative RT-PCR in Atlantic salmon is affected by viral infection. Vet Res 42:1–5 21314970 10.1186/1297-9716-42-8 PMC3031228

[CR34] Macqueen DJ, Robb DH, Olsen T, Melstveit L, Paxton CG, Johnston IA (2008) Temperature until the ‘eyed stage’of embryogenesis programmes the growth trajectory and muscle phenotype of adult Atlantic salmon. Biol Let 4:294–298 18348956 10.1098/rsbl.2007.0620 PMC2610038

[CR35] Medzhitov R (2001) Toll-like receptors and innate immunity. Nat Rev Immunol 1:135–145 11905821 10.1038/35100529

[CR36] Méré J, Chahinian A, Maciver SK, Fattoum A, Bettache N, Benyamin Y, Roustan C (2005) Gelsolin binds to polyphosphoinositide-free lipid vesicles and simultaneously to actin microfilaments. Biochem J 386:47–56 15527423 10.1042/BJ20041054 PMC1134765

[CR37] Morvan CL, Troutaud D, Deschaux P (1998) Differential effects of temperature on specific and nonspecific immune defences in fish. J Exp Biol 201:165–168 9405298 10.1242/jeb.201.2.165

[CR38] Pajdak-Czaus J, Platt-Samoraj A, Szweda W, Siwicki AK, Terech-Majewska E (2019) Yersinia ruckeri—A threat not only to rainbow trout. Aquac Res 50:3083–3096

[CR39] Paradis T, Bègue H, Basmaciyan L, Dalle F, Bon F (2021) Tight junctions as a key for pathogens invasion in intestinal epithelial cells. Int J Mol Sci 22:2506 33801524 10.3390/ijms22052506 PMC7958858

[CR40] Peterson R, Spinney H, Sreedharan A (1977) Development of Atlantic salmon (Salmo salar) eggs and alevins under varied temperature regimes. J Fish Board Canada 34:31–43

[CR41] Roberts R, Agius C, Saliba C, Bossier P, Sung Y (2010) Heat shock proteins (chaperones) in fish and shellfish and their potential role in relation to fish health: a review. J Fish Dis 33:789–801 20678104 10.1111/j.1365-2761.2010.01183.x

[CR42] Rozas-Serri M, Lobos C, Correa R, Ildefonso R, Vásquez J, Muñoz A, Maldonado L, Jaramillo V, Coñuecar D, Oyarzún C (2020) Atlantic salmon pre-smolt survivors of Renibacterium salmoninarum infection show inhibited cell-mediated adaptive immune response and a higher risk of death during the late stage of infection at lower water temperatures. Front Immunol 11:1378 32695119 10.3389/fimmu.2020.01378 PMC7338658

[CR43] Sahoo BR (2020) Structure of fish Toll-like receptors (TLR) and NOD-like receptors (NLR). Int J Biol Macromol 161:1602–1617 32755705 10.1016/j.ijbiomac.2020.07.293 PMC7396143

[CR44] Sanahuja I, Ibarz A (2015) Skin mucus proteome of gilthead sea bream: a non-invasive method to screen for welfare indicators. Fish Shellfish Immunol 46:426–435 26134830 10.1016/j.fsi.2015.05.056

[CR45] Scharsack JP, Franke F (2022) Temperature effects on teleost immunity in the light of climate change. J Fish Biol 101:780–796 35833710 10.1111/jfb.15163

[CR46] Scott GR, Johnston IA (2012) Temperature during embryonic development has persistent effects on thermal acclimation capacity in zebrafish. Proc Natl Acad Sci 109:14247–14252 22891320 10.1073/pnas.1205012109 PMC3435178

[CR47] Sommerset I, Nielsen JW, Oliveira VH, Moldal T, Bornø G, Haukaas A, Brun E (2023) Fish health report 2022. Report no. 5a/2023*.* Norwegian Veterinary Institute (NVI). https://www.vetinst.no/rapporter-og-publikasjoner/rapporter/2023/norwegian-fish-health-report-2022

[CR48] Tobback E, Decostere A, Hermans K, Ryckaert J, Duchateau L, Haesebrouck F, Chiers K (2009) Route of entry and tissue distribution of Yersinia ruckeri in experimentally infected rainbow trout Oncorhynchus mykiss. Dis Aquat Org 84:219–228 10.3354/dao02057 19565699

[CR49] Trosky JE, Liverman AD, Orth K (2008) Yersinia outer proteins: Yops. Cell Microbiol 10:557–565 18081726 10.1111/j.1462-5822.2007.01109.x

[CR50] Volkoff H, Rønnestad I (2020) Effects of temperature on feeding and digestive processes in fish. Temperature 7:307–320 10.1080/23328940.2020.1765950 PMC7678922 33251280

[CR51] Wentworth SA, Thede K, Aravindabose V, Monroe I, Thompson AW, Molyneaux N, Owen CL, Burns JR, Gonzalez-Vicente A, Garvin JL (2018) Transcriptomic analysis of changes in gene expression of immune proteins of gill tissue in response to low environmental temperature in fathead minnows (Pimephales promelas). Comp Biochem Physiol d: Genomics Proteomics 25:109–117 29414190 10.1016/j.cbd.2017.11.004

[CR52] Wildgoose W (1998) Skin disease in ornammental fish: identifying common problems. In Pract 20:226–243

[CR53] Wittmann J, Dieckow J, Schröder H, Hampel U, Garreis F, Jacobi C, Milczarek A, Hsieh K, Pulli B, Chen J (2018) Plasma Gelsolin Promotes Re-Epithelialization. Sci Rep 8:13140 30177722 10.1038/s41598-018-31441-2 PMC6120956

[CR54] Ytteborg E, Baeverfjord G, Torgersen J, Hjelde K, Takle H (2010) Molecular pathology of vertebral deformities in hyperthermic Atlantic salmon (Salmo salar). BMC Physiol 10:1–16 20604915 10.1186/1472-6793-10-12 PMC2914708

[CR55] Zhang Q, Kopp M, Babiak I, Fernandes JMO (2018) Low incubation temperature during early development negatively affects survival and related innate immune processes in zebrafish larvae exposed to lipopolysaccharide. Sci Rep 8:4142. 10.1038/s41598-018-22288-8 29515182 10.1038/s41598-018-22288-8 PMC5841277

