# Supplementary figures and images for: Embryonic Temperature Influences the Mucosal Responses of Atlantic Salmon Alevins to a Bacterial Challenge
Source: Mar Biotechnol (NY). 2024 Nov 19;27(1):1. doi: 10.1007/s10126-024-10386-w (PMC11576808; doi:10.1007/s10126-024-10386-w)

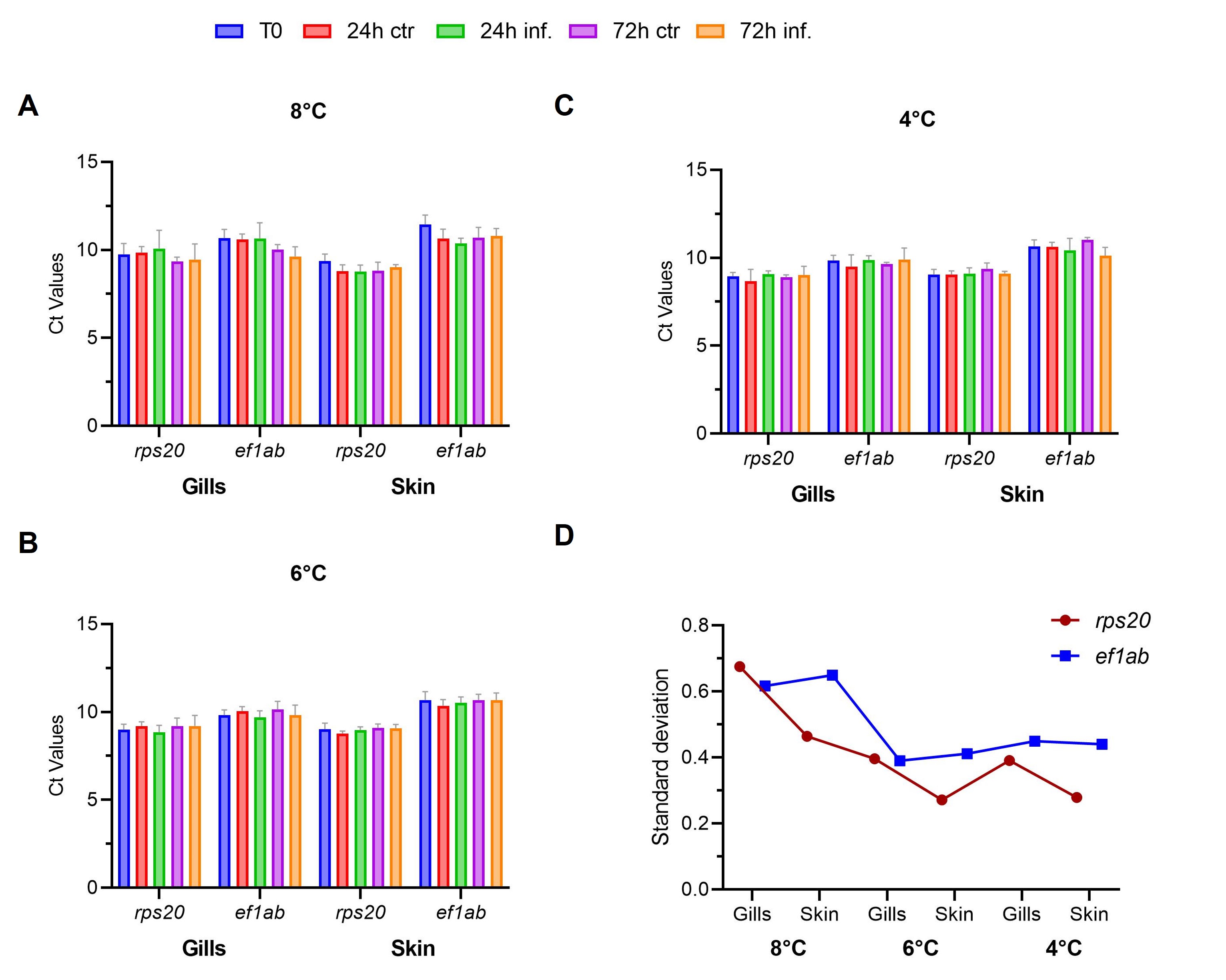

Supplement: Supplementary file 1 — Supplementary file1 (JPG 602 KB) [file 10126_2024_10386_MOESM1_ESM.jpg]

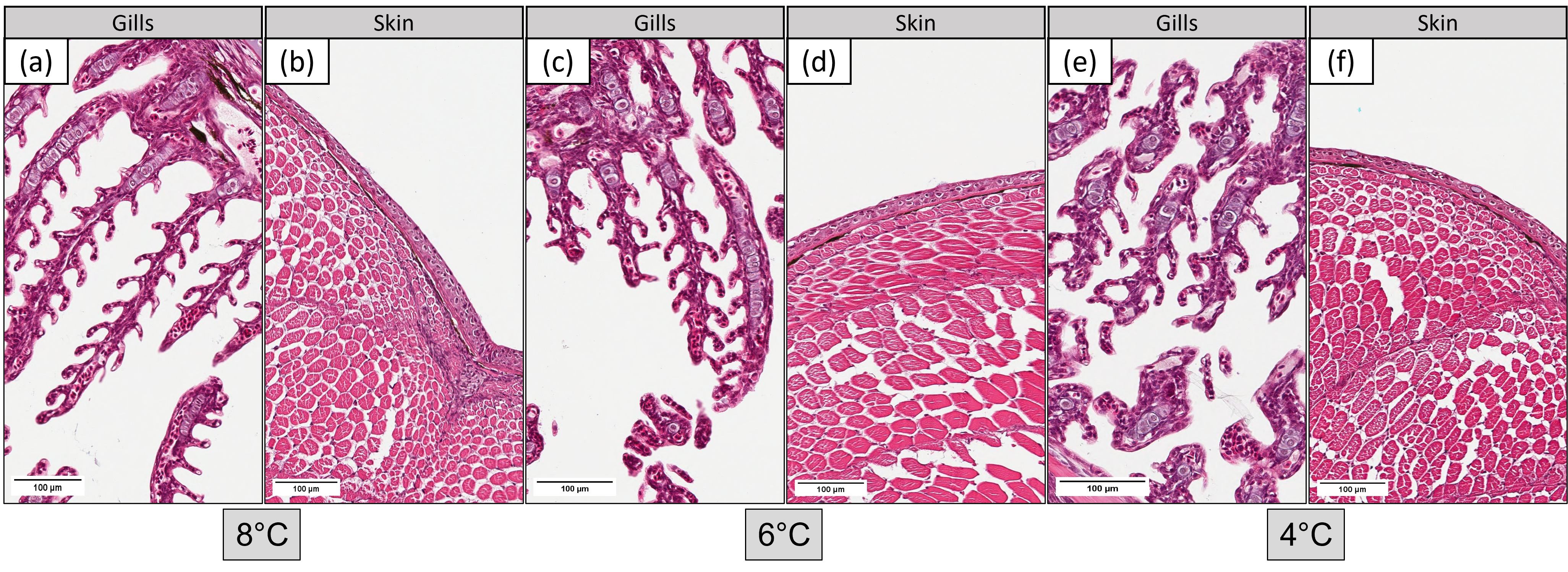

Supplement: Supplementary file 2 — Supplementary file2 (PNG 10809 KB) [file 10126_2024_10386_MOESM2_ESM.png]

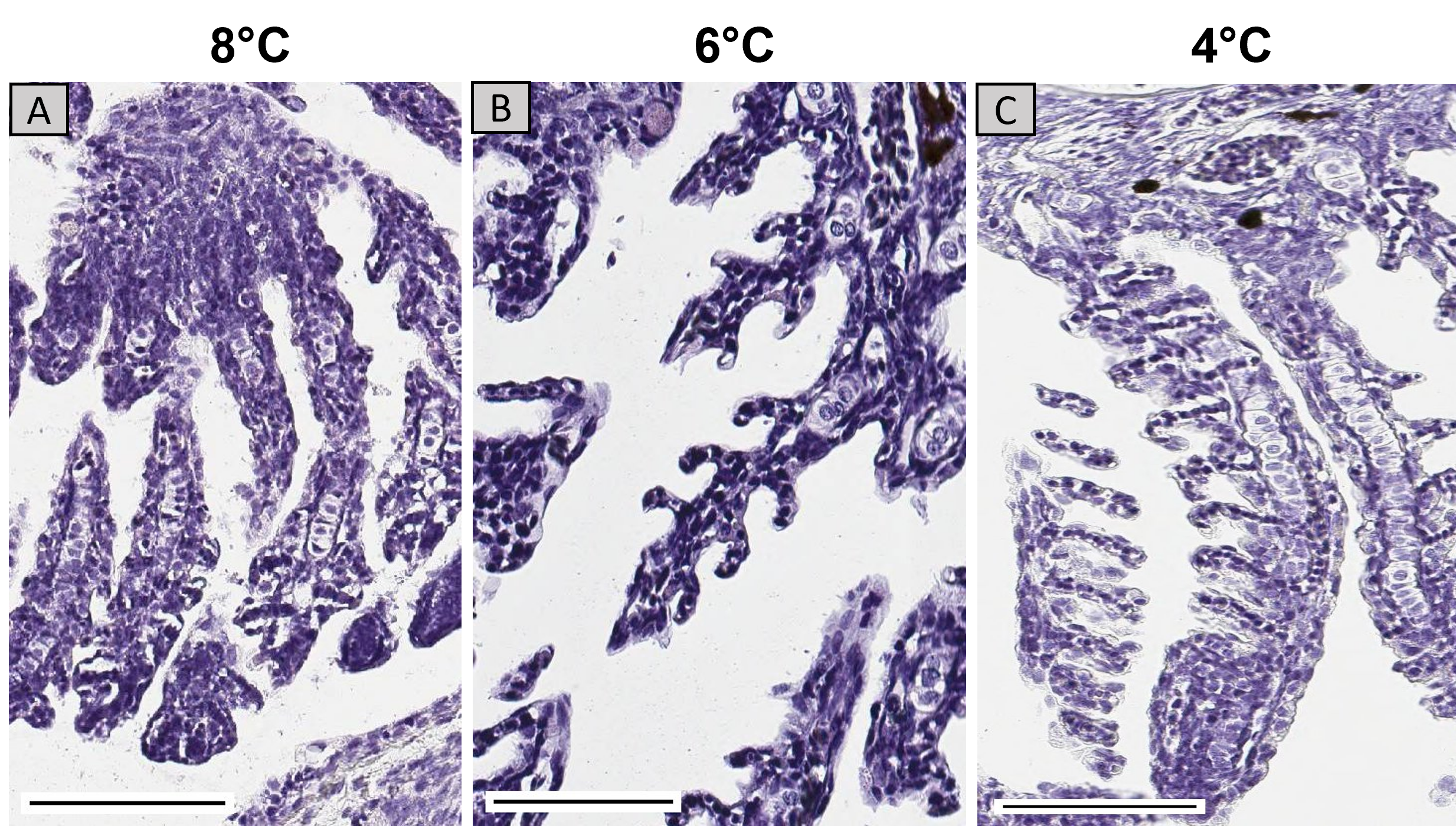

Supplement: Supplementary file 3 — Supplementary file3 (PNG 7487 KB) [file 10126_2024_10386_MOESM3_ESM.png]

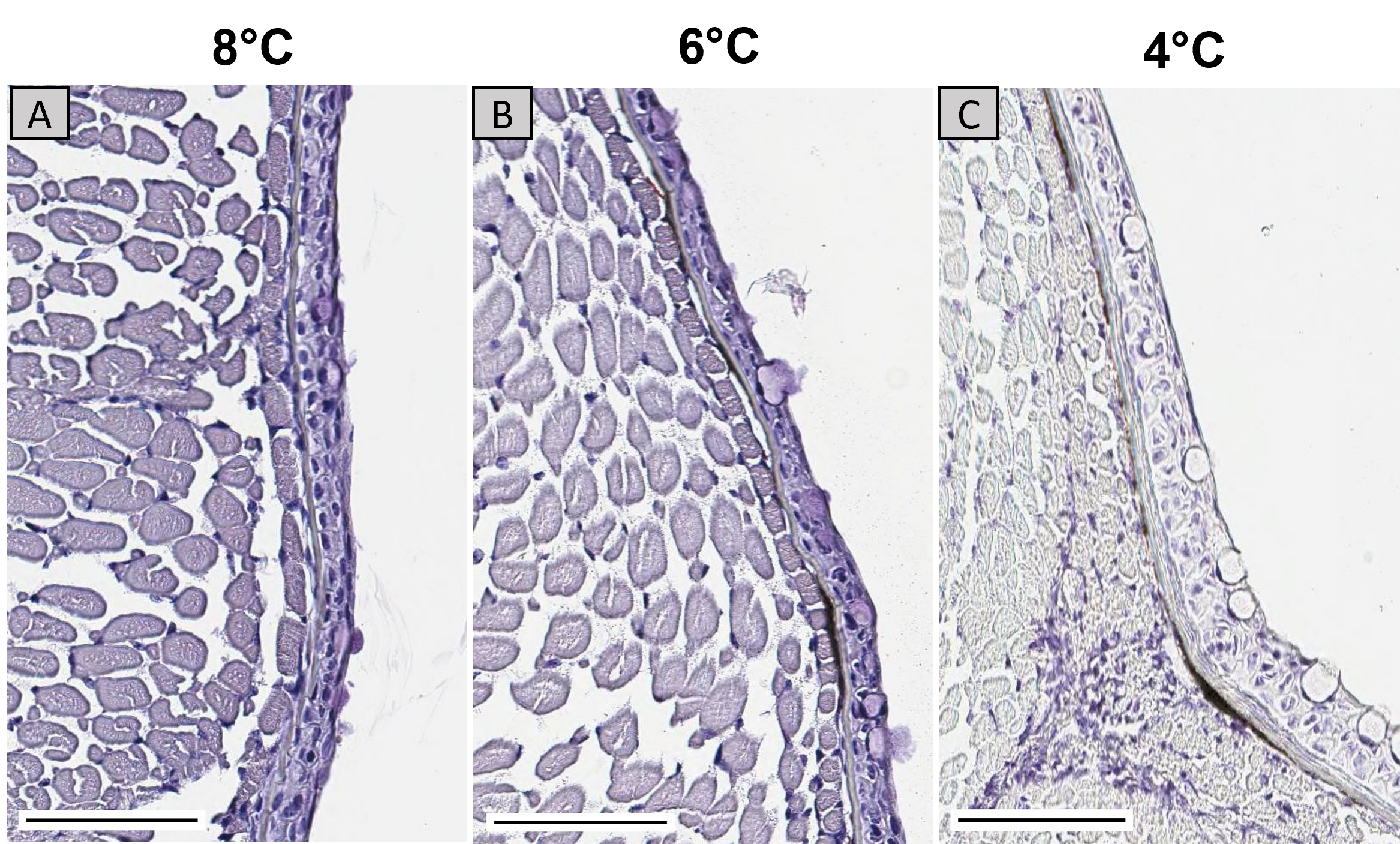

Supplement: Supplementary file 4 — Supplementary file4 (PNG 6344 KB) [file 10126_2024_10386_MOESM4_ESM.png]
